# 

**Published:** 1904-08

**Authors:** 


					﻿Uwenttetb Jl)ear.
Vol. XX.
AUGUST, 1904.
No. 2.J
TEXAS
Medical Journal.
k ^Kjbscri ption, $1.00 a Year, in Advance.
nr Single Copies, io Cents.
Of Flnp
PUILISHED AT AUSTIN, TEXAS, BY
I’. E. DANIEL, M. D.,
—....... Proprietor.
printed by;the*von boko km ANN-JO neb oo.
Entered at the Postoffice at Austin, Texas, as Heeond-class Mail Matter.
				

